# Diabetic ketoacidosis as the initial presentation of hepatogenous diabetes: a first reported case

**DOI:** 10.1530/EDM-25-0088

**Published:** 2026-02-17

**Authors:** Mhd Kutaiba Albuni, Shahem Abbarh, Noheir Ashraf Ibrahem Fathy Hassan, Bisher Sawaf, Ashraf I Ahmed, Yusuf Hallak, Muaataz Azzawi

**Affiliations:** ^1^ Internal Medicine Department, TriHealth Good Samaritan Hospital, Cincinnati, Ohio, USA; ^2^ Internal Medicine Department, MedStar Health Georgetown University, Baltimore, Maryland, USA; ^3^ Faculty of Medicine Aswan University, New Aswan City, Egypt; ^4^ Internal Medicine Department, University of Toledo Medical Center, Toledo, Ohio, USA; ^5^ Division of Internal Medicine, Hamad Medical Corporation, Doha, Qatar; ^6^ College of Medicine, QU Health, Qatar University, Doha, Qatar; ^7^ Gastroenterology Department, Southern Illinois University, Springfield, Illinois, USA

**Keywords:** hepatogenous diabetes, diabetic ketoacidosis, cirrhosis, guselkumab

## Abstract

**Summary:**

Hepatogenous diabetes, a secondary form of diabetes arising from chronic liver disease, particularly cirrhosis, is a well-documented complication. However, diabetic ketoacidosis (DKA) in the context of hepatogenous diabetes has not been reported in the literature. We report the case of a 60-year-old male with alcoholic liver cirrhosis and no prior history of diabetes who presented with altered mental status and was diagnosed with DKA. Initial lab tests revealed severe hyperglycemia, high-anion gap metabolic acidosis, and an elevated HbA1C of 10.2%, a significant increase from 5.2% five months earlier. The patient was managed with insulin and lactulose, resulting in clinical improvement. Follow-up revealed normalization of HbA1C and a reduction in insulin requirements. This is the first documented case of DKA as the initial presentation of hepatogenous diabetes, emphasizing the need for heightened awareness and further research into its clinical manifestations and management.

**Key clinical messages:**

- DKA can be the first clinical manifestation of hepatogenous diabetes, even in patients with normal HbA1C and no prior diabetes history.
- HbA1C is an unreliable marker for diagnosing diabetes in cirrhotic patients due to shortened red blood cell lifespan; OGTT should be considered when hepatogenous diabetes is suspected despite normal glycemic markers.
- Subclinical infections can precipitate DKA in cirrhosis through stress-induced insulin resistance.

## Introduction

A coordinated response of insulin secretion, hepatic and peripheral glucose absorption, and reduction in hepatic glucose production is required to maintain glucose homeostasis. The liver, pancreas, muscles, adipose tissues, and many circulating factors are among the many tissues and inter-organ crosstalk that contribute to this intricate regulatory mechanism (1). Through controlling many glucose metabolic processes, including glycolysis, glycogenolysis, gluconeogenesis, and glycogenesis, the liver plays a crucial part in maintaining glucose homeostasis (2). Thus, glucose metabolism is likely to be affected by hepatic dysfunction. Indeed, there has long been evidence linking diabetes mellitus (DM) to liver cirrhosis (LC) (3). To refer to DM brought on by LC, Naunyn originally used the term ‘hepatogenous diabetes’ (HD) in 1906 (4). Insulin resistance (IR), hyperinsulinemia, and pancreatic β-cell dysfunctions were often described as the relationship between DM and LC was examined in greater detail in the following years (5).

Diabetic ketoacidosis (DKA) is a fatal life-threatening condition whose symptoms include acidosis, hyperglycemia, and ketonemia. It affects mainly people with type 1 diabetes; however, those with type 2 diabetes can also have it. In both populations, catabolic stress of acute illness or injuries such as trauma, surgery, or infections may be a trigger. Common precipitating factors for DKA are non-compliance, new-onset diabetes, and other acute medical illnesses (6). Despite the fact that DKA is rare in HD, its occurrence emphasizes the necessity of careful monitoring and proper glucose control in cirrhosis patients (7). In this case report, we describe a case of acute-onset DKA in a patient with a history of alcoholic cirrhosis and no history of diabetes or pre-diabetes.

## Case presentation

### Case presentation and investigation

A 60-year-old male presented with a past medical history of psoriatic arthritis and alcohol-associated LC, complicated by portal hypertension and esophageal varices. The patient was brought to the emergency room with a 3-day history of altered mental status. Home medications include guselkumab 100 mg every 8 weeks, furosemide 40 mg daily, spironolactone 100 mg daily, carvedilol 25 mg twice daily, and lactulose 10 mL daily. Noteworthily, guselkumab was switched from adalimumab four months before presentation due to recurrent methicillin-sensitive staphylococcus aureus (MSSA) bacteremia secondary to lower limb cellulitis. He has no previous history of diabetes or pre-diabetes, with a last HbA1C of 5.2% five months before presentation. His current presentation was associated with malaise, fatigue, and decreased appetite. Later, he started to have abnormal behavior, such as fidgeting movements, mistaking the door for a window, and urinating on the floor. On physical examination, he was obtunded, responsive to painful stimulation only, and was able to protect his airways. Vital signs showed a blood pressure of 148/98 mmHg, a heart rate of 98 beats/minute, a respiratory rate of 22 breaths/minute, and a saturation of 98% on room air with a temperature of 98.8F. An urgent computed tomography (CT) scan of the head showed no acute insults. Abdominal ultrasound showed cirrhotic liver with mild ascites not amenable to tapping. Lab tests were significant for high-anion gap metabolic acidosis with a bicarbonate of 17 mEq/L, an anion gap of 16 mEq/L, a glucose level of 427 mg/dL, and a beta-hydroxybutyrate of 2.78 mmol/L. Interestingly, his repeated HbA1C was 10.2%. The rest of the lab results are summarized in Table 1.

**Table 1:** Summary of laboratory results.

Tests	Result	Normal range
Bicarbonate, mEq/L	17	22–28
Anion gap, mEq/L	16	8–12
Glucose, mg/dL	427	70–100
BHB, mmol/L	2.78	0.02–0.27
HbA1C, %	10.2	4.0–5.6
Sodium, mEq/L	126	135–145
AST, U/L	94	10–40
ALT, U/L	96	7–56
Total bilirubin, mg/dL	4.5	0.1–1.2
Creatinine, mg/dL	0.92	0.6–1.2
Urea, mg/dL	37	7–20
WBC, ×10^9^/L	11.4	4.0–11.0
Hb, g/dL	13	13–17 (male)
PLT, ×10^9^/L	131	150–400
C-peptide, ng/mL	1.93	0.5–2.0
Anti-insulin Ab	Negative	Negative
Anti-GAD Ab	Negative	Negative

AST, aspartate aminotransferase; ALT, alanine aminotransferase; Ab, antibodies; BHB, beta-hydroxybutyrate; WBC, white blood cell count; Hb, hemoglobin; PLT, platelets; GAD, glutamic acid decarboxylase.

## Treatment

The patient was admitted to the intensive care unit and started on lactulose and an insulin drip as per DKA protocol. He received a regular insulin drip at 0.1 units/kg/h for the first three hours, before transitioning to 0.05 units/kg/hr until he was bridged to subcutaneous insulin the next day. He gradually improved in the next two days and returned to his usual status. The patient was discharged on 100 units of long-acting insulin and 25 units of prandial insulin with each meal. A workup for his new-onset DM was sent and showed normal C-peptide levels (1.93 ng/mL) and negative antibodies, anti-insulin and anti-glutamic acid decarboxylase (GAD) antibodies. A CT scan of the abdomen showed cirrhosis with portal hypertension and upper abdominal varices with a normal pancreas.

## Outcome and follow-up

The patient did well after discharge. At the three-month follow-up, his HbA1C decreased to 5.8%. His insulin requirements have gradually decreased to 10 units of long-acting insulin only, and he has not required any hospital readmissions during the year following his discharge.

## Discussion

This case showed acute symptomatic hyperglycemia and DKA with metabolic acidosis in a cirrhotic patient with no previous history of DM or even pre-diabetes after switching to guselkumab. This case report lacked a clear explanation for the patient’s abrupt onset of DKA and highly elevated HbA1C; however, there are multiple theories to be discussed in trying to explain this sudden onset of DKA in this patient.

Targeting the IL-23p19 cytokine subunit, guselkumab is a monoclonal antibody that has been approved to treat moderate-to-severe psoriasis in adults (8). There were 24,312 reports of guselkumab-related adverse events found in the FAERS database. Diabetes was not particularly mentioned as a documented adverse event in the research, despite the fact that it highlighted a number of adverse occurrences, such as skin-related problems and cancers (9). When guselkumab was tested in individuals with moderate-to-severe psoriasis in the VOYAGE 1 and VOYAGE 2 trials, the safety profiles matched those seen in earlier research. Although there were a few significant adverse events, such as cardiovascular events, documented in the studies, the incidence of diabetes was not carefully addressed (10). Recurrent infections, headaches, weariness, injection site reactions, and diarrhea are among the side effects that are often reported (11). The patient’s prescription list had only changed once in the previous six months, and that was four months before the presentation when adalimumab was switched to guselkumab. In addition, even though he continued taking guselkumab, his HbA1C and insulin demand dropped, which suggests that it is unlikely to be the cause of this patient’s significant rise in HbA1C and presentation of DKA.

Adalimumab, an inhibitor of tumor necrosis factor-alpha (TNF-α) inhibitor, is a human-recombinant monoclonal antibody. Adalimumab is used to treat several autoimmune diseases, including psoriasis, rheumatoid arthritis, and ankylosing spondylitis (12). According to several clinical studies, adalimumab is often well tolerated. Serious side effects included hepatotoxicity, lymphomas, demyelinating diseases, pancytopenia, congestive heart failure, and infections (13). The effects of adalimumab on blood glucose levels were assessed in an experimental investigation using obese diabetic rats. According to the study, administering adalimumab significantly reduced fasting glucose levels, suggesting that it may have a therapeutic effect on blood glucose regulation (14). A practical pharmacovigilance analysis looked at adverse event records to find hypoglycemic indicators linked to TNF-α inhibitors. A strong disproportionality signal for hypoglycemia was found in the study of individuals using adalimumab, especially those with signs of ankylosing spondylitis, rheumatoid arthritis, and psoriasis (15). Desai *et al*. published a cohort study that examined the incidence of incident diabetes mellitus in rheumatoid arthritis patients receiving treatment with different biologic or targeted synthetic disease-modifying medications. Adalimumab-treated individuals were more likely to acquire diabetes mellitus than those receiving other drugs, according to the research (14). Wu & Tsai published a case report of a psoriasis patient who had recurrent hyperglycemia while using adalimumab. Once the medicine was stopped, the patient’s blood glucose levels stabilized. This raises the possibility of a medication-induced impact on glucose metabolism (16). However, this patient’s HbA1C was normal while he was on adalimumab and he was not diabetic or even pre-diabetic; also, he developed DKA four months after switching from adalimumab to guselkumab, indicating that adalimumab can be excluded as a cause of this patient’s DKA.

Cirrhosis is a chronic, progressive, and diffuse process characterized by fibrosis and the conversion of normal liver architecture into structurally abnormal nodules (17). The most common causes of cirrhosis in the United States are hepatitis C, alcohol-associated liver disease, and metabolic-dysfunction-associated steatotic liver disease (MASLD) (18). Type 2 diabetes was found to be independently associated with cirrhosis (19). Diabetes mellitus (DM) that develops as a consequence of LC is referred to as HD. In patients with LC, the prevalence rates of HD have been reported to vary from 21 to 57% (20). Previous research that used Taiwan’s National Health Insurance Research Database looked at the relationship between type 2 diabetes and LC. Patients with cirrhosis were shown to be more likely to develop type 2 diabetes than those without the condition, with those with alcoholic cirrhosis being at the highest risk (21). The importance of liver health in glucose metabolism was highlighted by another prospective cohort research that found fatty liver indices to be predictive of the incidence of new-onset diabetes over a 9-year period (22). A cross-sectional examination of 1,068 individuals with cirrhosis revealed that almost 40% of them had diabetes. The study showed that the development of diabetes was closely linked to cirrhosis etiology, including alcoholic liver disease and non-alcoholic steatohepatitis (NASH) (23). Glycemic impairment in cirrhosis patients has a complicated etiology. It is thought that pancreatic β-cell dysfunction and peripheral IR are important contributing causes. Hyperinsulinemia and downregulation of insulin receptors result from decreased insulin clearance caused by reduced liver function and portosystemic shunting (24). Furthermore, cirrhosis-related hypoxia, which is characterized by elevated levels of hypoxia-inducible factor-1 alpha (HIF-1α), is detrimental to β-cell function and contributes to the development of HD (25). In addition, it has been shown that cirrhosis resulting from hepatocyte injury is associated with increased levels of betatrophin, which is expressed by hepatocytes. Research has demonstrated a correlation between increased betatrophin and hepatic IR, as indicated by the homeostatic model assessment for insulin resistance (HOMA-IR) (26). Similarly, cirrhosis patients’ euglycemic clamp trials showed higher glucose levels despite similar stimulated insulin levels, suggesting that IR impairs skeletal muscle’s ability to absorb glucose (27). Similar to type 2 diabetes, HD is diagnosed and evaluated clinically using HbA1C, oral glucose tolerance test (OGTT), and fasting plasma glucose (FPG). However, because liver failure impairs hepatic glucose production, FPG may be less sensitive in individuals with liver illness. Similar to this, HbA1C should be read cautiously in patients with chronic liver disease since it may be underestimated in cirrhosis due to the shorter lifetime of the red blood cells caused by hypersplenism. Therefore, in cirrhosis patients with normal FPG and HbA1C, OGTT is required to diagnose diabetes mellitus or impaired glucose tolerance (28). Unlike classical type 2 diabetes, HD is less commonly associated with traditional risk factors, such as family history of diabetes, age, and body mass index. Moreover, patients with HD frequently present with abnormal OGTT results despite normal FPG and HbA1C (28); this may explain why this patient’s HbA1C was normal despite having cirrhosis for more than 10 years. Along with blood albumin, total bilirubin, prothrombin activity, and Child–Pugh score, OGTT was also demonstrated to be a useful predictive factor in cirrhosis patients (4). Although OGTT has not been performed lately to accurately test for HD or reduced glucose tolerance, the patient had cirrhosis for over a decade and was not recognized to have symptoms of diabetes prior to the current presentation.

An infection-induced DKA is a well-known clinical situation, especially among diabetics (6). However, infections that severely disturb insulin and glucose homeostasis can also cause DKA in persons who do not have diabetes. Acute pancreatitis led to the development of hyperglycemic ketoacidosis in a 70-year-old lady who had no history of diabetes. Upon discharge, she did not require insulin therapy because her HbA1C was normal (29). After presenting with COVID-19 symptoms, a 52-year-old lady who had no previous history of diabetes was diagnosed with DKA. She exhibited low levels of C-peptide and a normal HbA1C, both of which were indicative of newly diagnosed type 1 diabetes (30). A stress reaction brought on by an infection activates the hypothalamic–pituitary–adrenal axis, which raises cortisol, catecholamines (epinephrine and norepinephrine), glucagon, and growth hormone levels. These hormones counteract the effects of insulin, stimulate hepatic gluconeogenesis, glycogenolysis, and lipolysis, all of which raise blood sugar and free fatty acid levels and encourage the creation of ketone bodies (31). In systemic infections, elevated levels of pro-inflammatory cytokines, including TNF-α and IL-6, may damage pancreatic β-cells, suppress insulin production, induce IR, and enhance lipolysis/ketogenesis (31). An autoimmune reaction against pancreatic β-cells can occasionally be brought on by infections. This autoimmune response can cause insulin insufficiency and β-cell death, which can lead to DKA and new-onset diabetes (32). In individuals with LC, infections are a serious consequence that greatly increases morbidity and death. These infections frequently result from a confluence of increased intestinal permeability, gut dysbiosis, and compromised immune function (33). According to several research, the prevalence of spontaneous bacteremia in cirrhosis patients ranges from 4 to 21%. The occurrence of this syndrome is 10 times higher in cirrhotic individuals than in the general population. The severity of bloodstream infections in this group is shown by the death rate linked to these infections, which varies from 23 to 58% in cirrhotic individuals (34). Although this patient’s bacteremia has subsided few months before his presentation with DKA after switching to guselkumab, he might have had subclinical infection that has not been detected, or his bacteremia has not resolved completely, which may have led to his sudden onset of DKA due to stress response or immunomodulation.

## Conclusion

In the setting of HD, this case report demonstrates the uncommon and intricate presentation of DKA. HD is a serious but little-known side effect of cirrhosis and other chronic liver diseases. This is the first documented case of DKA as the initial sign of HD reported, which emphasizes how crucial it is to take secondary types of diabetes into account when cirrhosis patients exhibit hyperglycemia. Subclinical infection as a cause of sudden-onset DKA in cirrhotic patients should be suspected even with no history of diabetes. Future research is required to evaluate the incidence and pathogenesis of DKA in cirrhosis patients.

## Declaration of interest

The authors declare that there is no conflict of interest that could be perceived as prejudicing the impartiality of the work reported.

## Funding

This research did not receive a specific grant from funding agencies in the public, commercial, or not-for-profit sectors. Open access funding was provided by the Qatar National Library.

## Patient consent

A written informed consent was obtained from the patient for the publication of all images, clinical data, and other data included in the manuscript. All identifying information has been removed.

## Author contribution statement

MKA: conceptualization, methodology, visualization, writing – original draft, writing – review & editing. SA: validation, visualization, writing – original draft, writing – review & editing. NAIFH: validation, visualization, writing – original draft, writing – review & editing. BS: validation, visualization, writing – original draft, writing – review & editing. AIA: validation, visualization, writing – original draft, writing – review & editing. MA: validation, visualization, writing – original draft, writing – review & editing.
